# Identifying Anomalous Citations for Objective Evaluation of Scholarly Article Impact

**DOI:** 10.1371/journal.pone.0162364

**Published:** 2016-09-08

**Authors:** Xiaomei Bai, Feng Xia, Ivan Lee, Jun Zhang, Zhaolong Ning

**Affiliations:** 1 School of Software, Dalian University of Technology, Dalian 116621, China; 2 School of Information Technology and Mathematical Sciences, University of South Australia, Adelaide, South Australia, Australia; Universidad de las Palmas de Gran Canaria, SPAIN

## Abstract

Evaluating the impact of a scholarly article is of great significance and has attracted great attentions. Although citation-based evaluation approaches have been widely used, these approaches face limitations e.g. in identifying anomalous citations patterns. This negligence would inevitably cause unfairness and inaccuracy to the article impact evaluation. In this study, in order to discover the anomalous citations and ensure the fairness and accuracy of research outcome evaluation, we investigate the citation relationships between articles using the following factors: collaboration times, the time span of collaboration, citing times and the time span of citing to weaken the relationship of Conflict of Interest (COI) in the citation network. Meanwhile, we study a special kind of COI, namely suspected COI relationship. Based on the COI relationship, we further bring forward the COIRank algorithm, an innovative scheme for accurately assessing the impact of an article. Our method distinguishes the citation strength, and utilizes PageRank and HITS algorithms to rank scholarly articles comprehensively. The experiments are conducted on the American Physical Society (APS) dataset. We find that about 80.88% articles contain contributed citations by co-authors in 26,366 articles and 75.55% articles among these articles are cited by the authors belonging to the same affiliation, indicating COI and suspected COI should not be ignored for evaluating impact of scientific papers objectively. Moreover, our experimental results demonstrate COIRank algorithm significantly outperforms the state-of-art solutions. The validity of our approach is verified by using the probability of Recommendation Intensity.

## Introduction

Effective evaluation of a scholarly article has been an important research topic, as academic promotions and research grants assessment typically have significant weights towards the impacts of publication records. Unfortunately, anomalous citation activities do exist in practice, and the impacts of scholarly articles can be manipulated [1]. For example, some journals manipulate their high-impact status by means of self-citation and stack-citation [2]. Meanwhile, most of the impact evaluation methods for scholarly article do not account for anomalous citations [3, 4], possibly due to the difficulty of identifying diversified practices in anomalous citations. Thus, fair assessment of a scholarly article has been a challenging task in research performance evaluation.

For a long time, researchers in the academia have practiced impact evaluation of scholarly articles with bibliometrics and scientometrics. Citation count has been frequently used as the measure of the article’s scientific impact [5, 6]. Furthermore, many citation-based metrics have been proposed, such as the H-index [7, 8], the g-index [9], the impact factor (IF) [10], and the Eigenfactors scores [11]. Diverse ranking algorithms based on network topological structure sprung up to assess scholarly articles’ impact in recent years [3, 12–15]. MutualRank jointly ranked papers, authors, venues [14]. Futurerank algorithm estimated the expected future prestige scores of articles by comprehensively considering citation, authorship, and publication date features [15]. CAJTRank was proposed to accurately assess scientific articles by exploiting citation, authors, journals and time information [3]. Above four factors are the reason of its naming. MRFRank ranked the future importance of papers and authors together by using text features, time-aware weighted citation graph and co-authors graph [16]. A nonlinear PageRank algorithm was proposed to improve the effectiveness of ranking [17], with the high-score citing papers are favored and the low-score citing papers are punished.

Few studies have been conducted on distinguishing different citation relationships. Wan et al proposed the regression-based method, which uses a strength value to assess the importance of each citation according to several useful features. These features include occurrence number, located section, time interval, average length of citing sentences, average density of citation occurrences, and self-cited or not [18]. This method made a preliminary attempt to distinguish citation, and the effectiveness of citation strength distinction has also been demonstrated by its evaluation results. Valenzuela et al studied a simple citation distinction method, and proposed a supervised classification approach to identify important citations in scholarly publications. In that model, citations were simply classified into important and incidental citations. Citations appearing in the section of Methods or Discussions were considered as important citations, while citations appearing in Related Work part were considered as incidental citations [19]. The impact of scientific outputs was quantified by identifying positive and negative citations [20]. Self-citation was presented in different ways, including direct, co-author, collaborative and coercive induced self-citation [21]. In addition, there were some other related literatures such as the relationship analysis between self-citation and H-index [22], between self-citation and impact factor [23].

While the above-mentioned methods suggests citations may exhibit different importance, they do not explicitly reveal Conflict of Interest (COI) relationships in the citation network. In brief, COI indicates the person or organization sharing similar interests in various aspects, and they may cite the work conducted by themselves or by the people with close relationship. In reality, when two authors collaborate with each other, they are more likely to cite the work by one another. That means, anomalous citations may happen between co-authors because they are more familiar with each other not only in research, but also in person. To fairly and accurately assess the impact of a scholarly publication, it is necessary to weaken the effect of COI relationship. At the same time, even if two authors have not collaborated with each other, they may also anomalously cite each other for some reasons. For example, given two authors from the same affiliation, though they never collaborated with each other, they may deliberately cite each other because they are co-workers. We define this phenomenon as suspected COI relationship.

**Fig 1:**
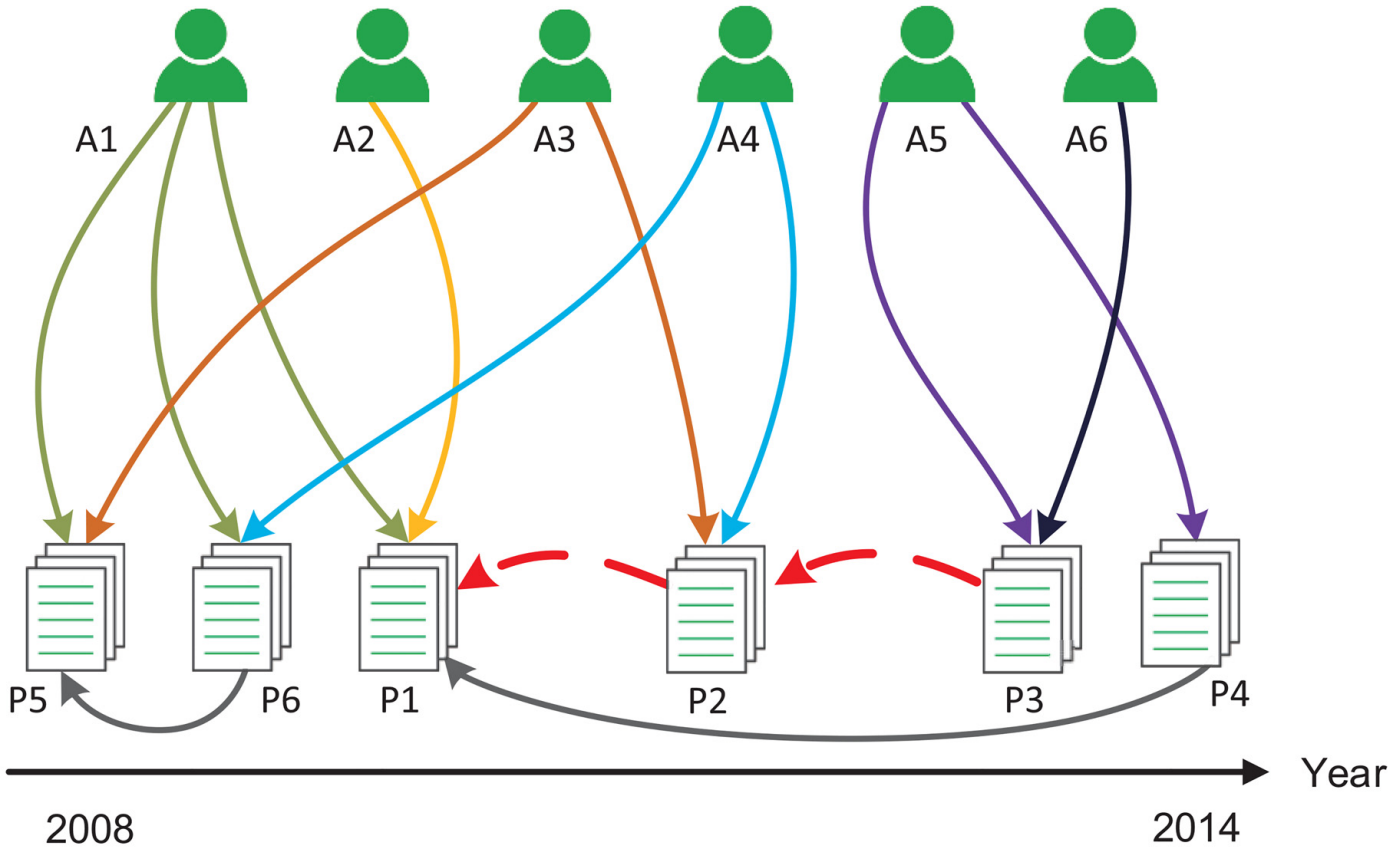
Illustrative example of COI relationship between different authors. Where *P*_*n*_ and *A*_*m*_ are the list of articles and authors respectively, red dashed line indicates citing relationship. The figure shows two cases: (1) Before *P*_*i*_ cites *P*_*j*_, the author(s) of *P*_*i*_ has (have) collaborated with the author(s) of *P*_*j*_, just like *P*_2_ cites *P*_1_, Author *A*_1_ and Author *A*_3_ co-author *P*_5_, Author *A*_1_ and Author *A*_4_ co-author *P*_6_, that is to say, there are two co-author pairs (*A*_1_, *A*_3_), (*A*_1_, *A*_4_); (2) Before *P*_*i*_ cites *P*_*j*_, the author(s) of *P*_*i*_ has (have) not collaborated with the author(s) of *P*_*j*_, just like *P*_3_ cites *P*_2_, however, if author *A*_3_ and author *A*_5_ belong to the same affiliation, (*A*_3_, *A*_5_) composes suspect COI author pair.

In this paper, we primarily focus on two issues: (1) identifying the COI relationship and distinguishing the strength of citation relationship; and (2) leveraging the strength of citation relationship to evaluate the impact of scholarly article by a mutual reinforce mechanism. An example of the COI relationship is shown in Fig 1. Modified PageRank (web Page Rank) and HITS (Hyperlink-Induced Topic Search) [24] are utilized in the proposed model. The main novelty of our algorithm is that COI relationship and suspected COI relationship are employed to quantify the citation strength of the articles. We leverage the following four factors: times of collaboration which is exploited to define the cooperation importance [25], time span of collaboration, times of citing and time span of citing for the measurement of COI relationship between researchers. We conduct extensive experiments on the Physical Review C (PRC) dataset, which is a subset of the APS. The results demonstrate that our method outperforms the existing approaches in Recommendation Intensity (RI) of list R at top-K, and we find that disclosing different citation relationship is significant to ensure the fairness and accuracy for evaluating the impact of scholarly articles. Furthermore, our solution has good compatibility with the existing citation-based metrics, such as IF, H-index, and g-index. In the subsequent section, we will describe our method that can quantify the scientific impact based on COI relationship in the citation network.

## Materials and Methods

In most previous impact evaluation work, all citation weightings are set as 1, which neglects the impact of COI and suspected COI. To address the issues of anomalous citations, we introduce the COIRank (Conflict of Interest-based Rank) algorithm which uses the COI relationship to distinguish the citing strength. And then, based on CAJTRank, we present an objective evaluative method to study the influence of scholarly manuscripts. The score of each manuscript is depicted by three kinds of information: citations, authors and journals. In this algorithm, we modify the PageRank algorithm, conduct the weighting processing, and distinguish different authors’ contributed scores for each article.

### Definition

In order to illustrate COIRank algorithm, a few concepts are defined below.

**COI relationship**: Given two types of entity sets *A* and *P*, where *A* = {*A*_1_, *A*_2_, …, *A*_*m*_}, and *P* = {*P*_1_, *P*_2_, …, *P*_*n*_}. Set *A* and set *P* represent the list of authors and papers, respectively. If authors *A*_*x*_ and *A*_*y*_ are co-authors in paper *P*_*k*_, subsequently paper *P*_*i*_ signed author *A*_*x*_ cites paper *P*_*j*_ signed author *A*_*y*_, we consider that potential COI relationship exists between authors *A*_*x*_ and *A*_*y*_. The definition aims to give less citing weight between papers since their authors are ever co-authors relationship.

**Suspected COI relationship**: Similar to the aforementioned definition of COI, definition of suspected COI aims to weaken the citing weight within same affiliation. Likewise, although authors *A*_*x*_ and *A*_*y*_ have not co-authored any paper, they belong to the same affiliation, then the relationship of author *A*_*x*_ and *A*_*y*_ is considered as suspected COI relationship.

**Citation relationship strength**: Citation can be distinguished by weakening COI in citation network. The citation relationship strength of an article citing another article is quantified by a numerical value, and the larger value represents the higher citation relationship strength, otherwise, represents the lower citation relationship strength. In this paper, the citation relationship strength is time-varying.

### Dataset

Our dataset consists of 41,751 authors and 30,966 publications from the Physical Review C (PRC), spanning over 43 years (from 1970 to 2013). 26,366 publications have been cited by other articles. The information of each article in the dataset includes its title, DOI, author(s), date of publication, affiliation(s) and publisher. In order to disclose the COI relationship in the citation network to fairly assess the impact of scholarly manuscripts, we firstly extract citations of PRC from the whole APS dataset. Then, for each two articles with existing citation relationship, we extract the co-author COI relationship and the suspected COI relationship before the citation happens to construct a weighted citation network.

### COIRank Algorithm

**Fig 2:**
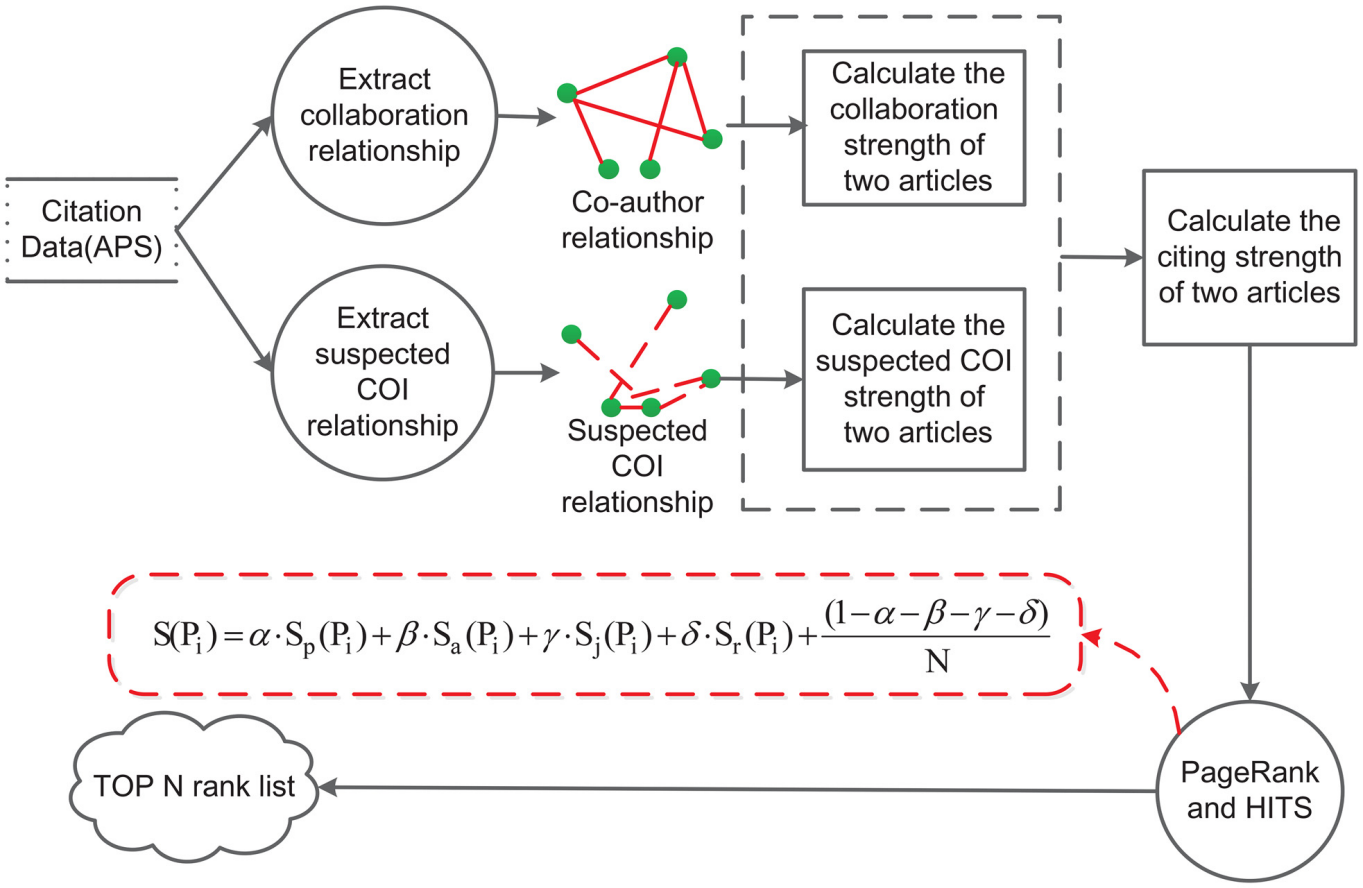
The structure of the COIRank algorithm. Including two steps: (1) Identifying COI and suspected COI; and (2) Generating the top *N* ranked list.

Based on the idea of CAJTRank [3], which ranks scholarly publications according author, publication, venue, and time information, we propose the COIRank method in this paper. As illustrated in Fig 2, the process of the COIRank algorithm is divided into two steps:

1. **Identifying COI and suspected COI**: The co-author relationship based on the number and the time span of collaborations is extracted; likewise, suspected COI relationship based on the number and the time span of citation is also extracted. Five scholarly metrics are used to compute the strength of citation relationship, which are utilized to guide the random walk in the citation network.
2. **Generating the top *N* ranked list**: we rank the scholarly articles by the mutual reinforce mechanism of PageRank and HITS algorithms. When COIRank ends, top *N* most influential articles are identified, and the result better reflects the influence of research manuscripts.

Details of these steps are further elaborated in the subsequent sections.

#### COI identification and citation relationship analysis

This section shows details of the five metrics used in our study, including: the strength of the co-author COI relationship, the strength of each two articles based on COI of co-authors, the strength of suspected COI relationship of each two authors, the strength of suspected COI relationship of each two articles, and the strength of citing relationship. Furthermore, we will present the details of COIRank based on the strength of citation relationship. To determine the citing strength, we consider two cases if Paper *P*_*i*_ cites Paper *P*_*j*_. In the first case, the authors of Paper *P*_*i*_ and Paper *P*_*j*_ have collaborated with each other before. In order to identify such a relationship, our studies assume that authors meeting all of the following criteria will be limited and the potential errors would be limited: (1) with the same name, (2) belongs to the same affiliation, and (3) work on the same research fields. In the second case, there exists no collaboration between authors of Paper *P*_*i*_ and Paper *P*_*j*_, while, the authors of Paper *P*_*i*_ and Paper *P*_*j*_ belong to the same affiliation. The former is direct COI which represents collaboration relationship, and the latter is indirect COI relationship which represents the relationship of suspected COI. If two authors collaborate a publication, there will be a link between them, and they may collaborate many times. Therefore, it is necessary to take the times of collaboration and time span of collaboration into account when measuring the collaboration strength between two authors. Accordingly, we use the number of citations and the time span of citation to distinguish the strength of suspected COI relationship of each two authors.

#### Strength of the co-author COI relationship

If author *A*_*x*_ and author *A*_*y*_ have co-author relationship before author *A*_*x*_ cites author *A*_*y*_, The COI strength of each two co-authors is defined as follows:

$$\begin{array}{c} W_{x,y}^{A-COI}=\frac{N_{x,y}^{Co-author}}{\Delta T_{c}} \end{array}$$ (1)

where $N_{xy}^{Co-author}$ represents the cumulative number of papers coauthored by the *x*th author and the *y*th author. $\Delta T_{c}=T_{x,y}^{N}-T_{x,y}^{1}+1$ indicates the number of year between the first and the last collaborations of authors *A*_*x*_ and *A*_*y*_. $W_{x,y}^{A-COI}$ denotes the COI strength of each two co-authors, which is the ratio between $N_{xy}^{Co-author}$ and Δ*T*_*c*_.

#### Strength of each two articles based on COI of co-authors

The relationship strength of each two articles based on the COI of co-author relationship is calculated by:

$$\begin{array}{c} W_{i,j}^{P-COI}=\sum_{x=1}^{X}\sum_{y=1}^{Y}\left(\frac{N_{x,y}^{Co-author}}{\Delta T_{c}}\right) \end{array}$$ (2)

where *X* and *Y* are the authors’ total numbers of a citing paper and a cited paper, respectively. Both *x* and *y* are initialized as 1, *x* indicates the author of a citing paper, and *y* indicates the author of a cited paper. $W_{ij}^{P-COI}$ denotes the COI strength of the *i*th paper and the *j*th paper, which is a cumulative sum of the COI strength of each two co-authors between the citing paper and the cited paper.

#### Strength of suspected COI relationship: authors

If authors *A*_*x*_ and *A*_*y*_ have never collaborated previously, and they belong to the same affiliation before author *A*_*x*_ cites author *A*_*y*_’s work, the strength of the suspected COI relationship of each two authors is formulated as follows:

$$\begin{array}{c} W_{x,y}^{A-SCOI}=\frac{N_{x,y}^{Cite}}{\Delta T_{s}} \end{array}$$ (3)

where $N_{xy}^{Cite}$ is the cumulative number of papers of the *x*th author citing the *y*th author. $\Delta T_{s}=T_{x,y}^{N}-T_{x,y}^{1}+1$ indicates the number of years between the first and the last citing of authors *A*_*x*_ and *A*_*y*_. $W_{xy}^{A-SCOI}$ denotes the suspected COI strength of the *x*th author and the *y*th author, which is the ratio between $N_{x,y}^{Cite}$ and Δ*T*_*s*_.

#### Strength of suspected COI relationship: articles

The strength of suspected COI relationship of each two articles is calculated by:

$$\begin{array}{c} W_{i,j}^{P-SCOI}=\sum_{x=1}^{X}\sum_{y=1}^{Y}\frac{N_{x,y}^{Cite}}{\Delta T_{s}} \end{array}$$ (4)

where $W_{i,j}^{P-SCOI}$ denotes the strength of suspected COI relationship between each two articles. It is a total sum of suspected COI strength of the *x*th author and the *y*th author between citing paper and cited paper.

According to the Futurerank algorithm [15], an exponentially decaying function is used to model the strength of citation relationship between paper *P*_*i*_ and paper *P*_*j*_. $W_{i,j}^{P-Cite}$ is defined within a range from 0 to 1. The reason behind is that the previous work assumes the citation strength as 1 without regard to the COI relationship.

#### Strength of citing relationship

If the relationship is considered, the value is reasonable in range (0 − 1]. If the authors of paper *P*_*i*_ citing paper *P*_*j*_ have not only collaboration, but also suspected COI relationship before citing, *W*_*i*,*j*_ will be 0, and $W_{i,j}^{P-Cite}$ will be 1, otherwise, $W_{i,j}^{P-Cite}$ will be between 0 and 1, meanwhile the value of *W*_*i*,*j*_ is $W_{i,j}^{P-COI}$ or $W_{i,j}^{P-SCOI}$. When the authors of a citing paper and a cited paper have a collaboration relationship, *W*_*i*,*j*_ is $W_{i,j}^{P-COI}$. When the authors of a citing paper and a cited paper have a suspected COI relationship, *W*_*i*,*j*_ shows $W_{i,j}^{P-SCOI}$. The formula will be at advantage for current citation. The strength of citation relationship of each two articles is formulated as follows:

$$\begin{array}{c} W_{i,j}^{P-Cite}=e^{-\rho \left(T^{Current}-T^{Cite}+1\right)W_{i,j}} \end{array}$$ (5)

where *T*^*current*^ is the current time, *T*^*Cite*^ is the time of paper *P*_*i*_ citing paper *P*_*j*_, *T*^*Current*^ − *T*^*Cite*^ + 1 is the number of years since paper *P*_*j*_ was cited by paper *P*_*i*_. *ρ* is a constant value to represent predefined decay parameter.

#### Generating the top *N* ranked list

We adopt PageRank and HITS to calculate the prestige scores of each scholarly article, authors and journals. The ranking procedure is conducted as follows:

1. All authority scores of publications are set as 1/*N*, where *N* indicates the number of all publications used for the study.
2. Calculate the scores of PageRank of papers in the citation network, the weight is the strength of citation relationship of each two scholarly manuscripts.
3. Calculate the scores of authors of each publication using HITS algorithm in the paper-author network, the weight is set according to the sequence of the authors.
4. Calculate the scores of journals of each publication using HITS algorithm in the paper-journal network.
5. Calculate the scores of references of each publication using HITS algorithm in the citation network.
6. Update the authority scores of publications according to the scores of PageRank, authors, the journal and the reference.
7. Repeat steps 2-6 until convergence is encountered.

#### Scores of PageRank

The score of PageRank of paper *P*_*i*_, *S*_*p*_(*P*_*i*_), is calculated by the citation network:

$$\begin{array}{c} S_{p}\left(P_{i}\right)=\sum_{P_{j}\in IN\left(P_{i}\right)}\frac{W_{j,i}}{\left|OUT(P_{j})\right|}S\left(P_{j}\right) \end{array}$$ (6)

where *IN*(*P*_*i*_) includes all the papers which link to paper *P*_*i*_, |*OUT*(*P*_*j*_)| is the total number of papers which link out from paper *P*_*j*_. *S*(*P*_*j*_) refers to the original score of paper *P*_*j*_ before iteration is updated. *W*_*j*, *i*_ illustrates the strength of the citation relationship of paper *P*_*j*_ citing paper *P*_*i*_.

#### Scores of authors

The scores of author(s) of each article, *S*_*a*_(*P*_*i*_), are calculated by HITS algorithm. The formula is as follows:

$$\begin{array}{c} S_{a}\left(P_{i}\right)=\frac{1}{T(A)}\cdot \sum_{A_{j}\in Neighbor\left(P_{i}\right)}\frac{\sum_{P_{k}\in Neighbor\left(A_{j}\right)}S\left(P_{K}\right)}{|Neighbor\left(A_{j}\right)|\cdot Sequence\left(P_{i},A_{j}\right)} \end{array}$$ (7)

where *T*(*A*) denotes a total score transmitted from all the authors to papers. *Neighbor*(*P*_*i*_) denotes the author list fitting in with paper *P*_*i*_, *Neighbor*(*A*_*j*_) is the set of papers of author *A*_*j*_, *S*(*P*_*K*_) denotes authority score of paper *P*_*K*_, |*Neighbor*(*A*_*j*_)| is the number of papers in this set. *Sequence*(*P*_*i*_, *A*_*j*_) is the position of author *A*_*j*_ in the author list of *P*_*i*_.

#### Scores of journals

The authority score of journal of each article, *S*_*j*_(*P*_*i*_), is calculated by the HITS algorithm. The formula is as follows:

$$\begin{array}{c} S_{j}\left(P_{i}\right)=\frac{1}{T(J)}\cdot \sum_{J_{j}\in Neighbor\left(P_{i}\right)}\frac{\sum_{P_{k}\in Neighbor\left(J_{j}\right)}S\left(P_{K}\right)}{\left|Neighbor(J_{j})\right|} \end{array}$$ (8)

where *T*(*J*) denotes total scores transferred from all the journals to papers, *Neighbor*(*P*_*i*_) is the journal that paper *P*_*i*_ published, and each paper has only one journal. *Neighbor*(*J*_*j*_) is the set of papers published on journal *J*_*j*_, |*Neighbor*(*J*_*j*_)| is the number of papers in *Neighbor*(*J*_*j*_).

#### Scores of references

The score of references of each article, *S*_*r*_(*P*_*i*_), is also calculated by the HITS algorithm. The formula is demonstrated as follows:

$$\begin{array}{c} S_{r}\left(P_{i}\right)=\frac{1}{T(P)}\cdot \sum_{P_{j}\in Neighbor\left(P_{i}\right)}\frac{\sum_{P_{k}\in Neighbor\left(P_{j}\right)}S\left(P_{K}\right)}{\left|Neighbor(P_{j})\right|} \end{array}$$ (9)

where *Reference*(*P*_*i*_) represents the score of paper *P*_*i*_ collected from hub papers in the citation network, *T*(*P*) is the total scores from all the hub papers. *Neighbor*(*P*_*j*_) is the set of papers which *P*_*j*_ links to, that is to say, *Neighbor*(*P*_*j*_) is the set of references of *P*_*j*_, |*Neighbor*(*P*_*j*_)| is the number of references of paper *P*_*j*_.

#### Authority scores

The authority scores of each manuscript include the manuscript which has obtain the score from other manuscripts that cite the manuscript, authors, journals and references of this work. The specific calculation formula is shown as:

$$\begin{array}{c} S\left(P_{i}\right)=\alpha \cdot S_{p}\left(P_{i}\right)+\beta \cdot S_{a}\left(P_{i}\right)+\gamma \cdot S_{j}\left(P_{i}\right)+\delta \cdot S_{r}\left(P_{i}\right)+\frac{1-\alpha -\beta -\gamma -\delta }{N} \end{array}$$ (10)

In our algorithm, the initial score of each research manuscript is set to be 1/*N*. When the current and previous scores of each manuscript are less than 0.0001, this iterative algorithm converges. *S*(*P*_*i*_) represents the authority score of paper *P*_*i*_. *α*, *β*, *γ* and *δ* are constant parameters which range between 0 and 1. We set the probability of random jump to 0.15 experimentally, and then, *α* + *β* + *γ* + *δ* = 0.85.

## Results

**Fig 3:**
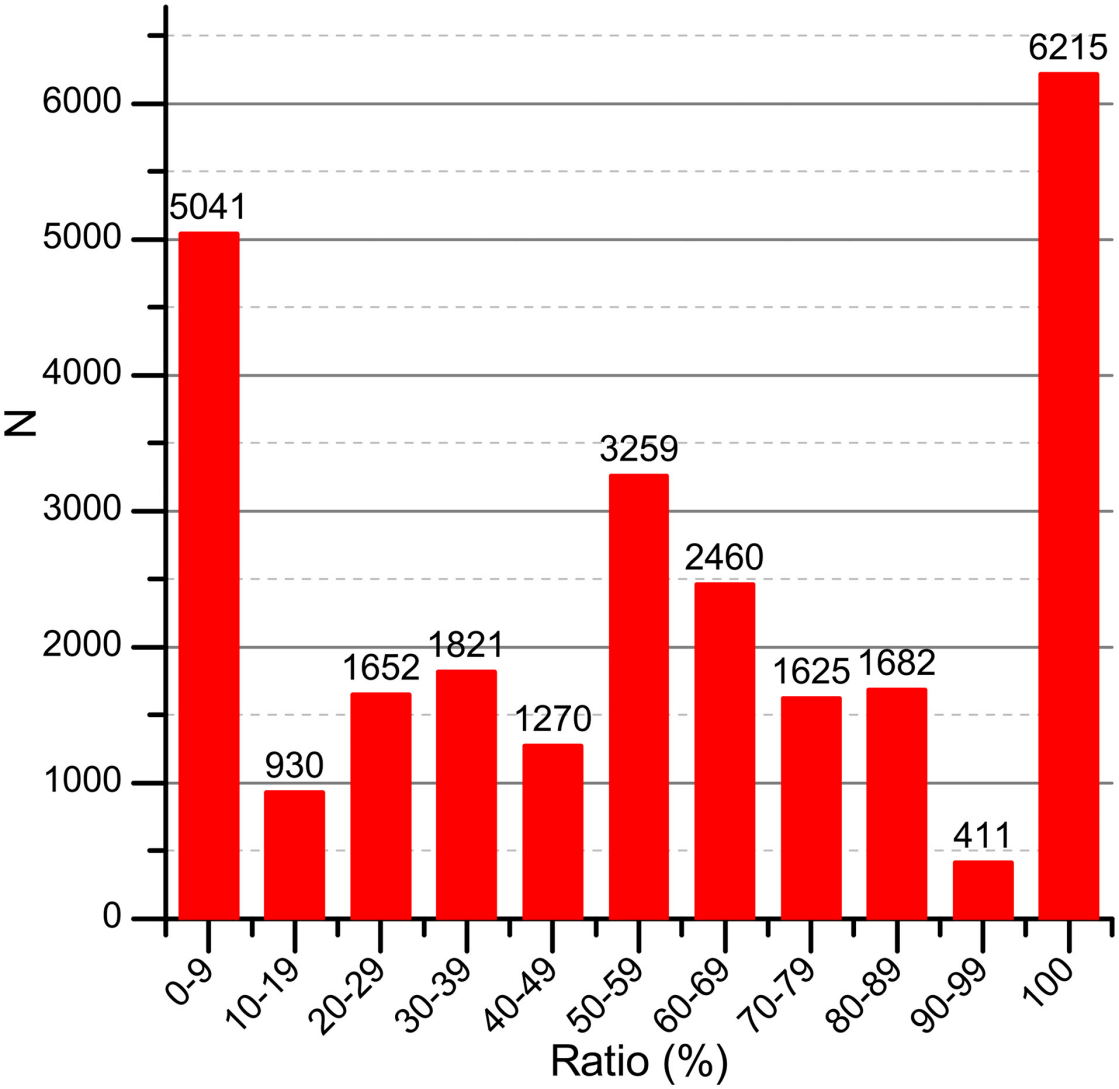
The ratio between contributed citations by co-authors and raw citations. The x-axis is the percentage between contributed citations by co-authors and total citations. The y-axis shows the number of articles.

Our work aims to provide an improved assessment of scientific output. In our first study, we examine the COI relationships exhibited in an existing journal. Out of 30,966 publications collected in the dataset, 26,366 publications attract one or more citations from other manuscripts. 21,324 publications have been cited by their co-authors, i.e. self-citations. 6,215 publications (or 23.57%) only have self-citations, as shown in Fig 3. In our next study, we investigate the citation behaviors of authors from the same affiliation. We observe that 19,920 publications have been cited by the authors belonging to the same affiliation, and 3,783 publications attract citations completely from the same affiliation as the author(s). Citations like the cases above could inflate the scholarly impact of one manuscript, which affects the impact of scholars and journals as well.

**Fig 4:**
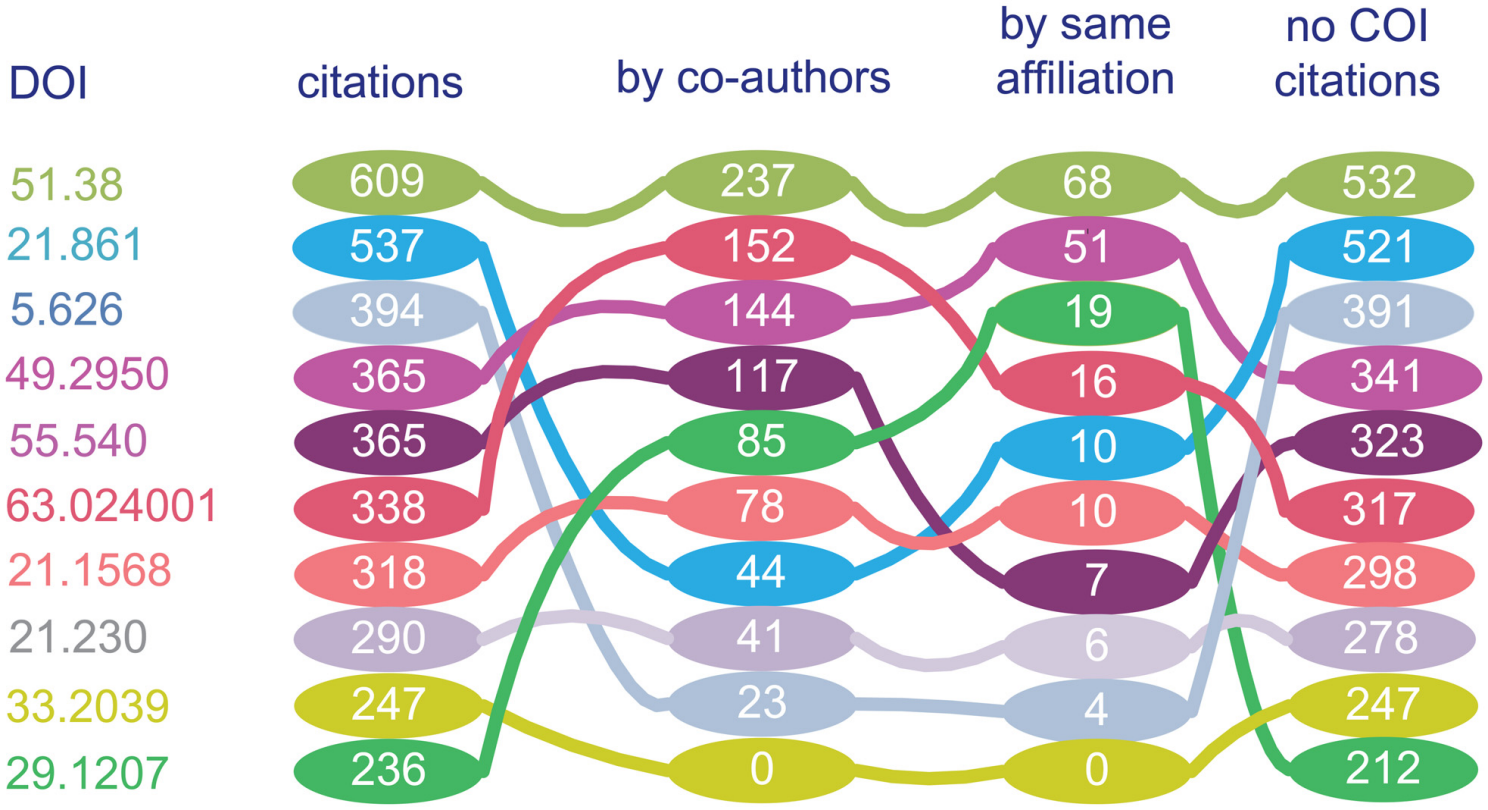
Ten most cited articles in the PRC dataset. It demonstrates the sources of citations and comparison between different rankings. DOI of ten articles omits the same fraction “10.1103/PhysRevC.”. The contributed citations by co-authors of the paper are different as we can observe. Compared with the co-author, the same affiliation(s) with author(s) also has a certain contribution to the citations. The COI phenomenon is very serious in the citation network. By weakening the strength of citing, we may obtain the citations without COI.

**Fig 5:**
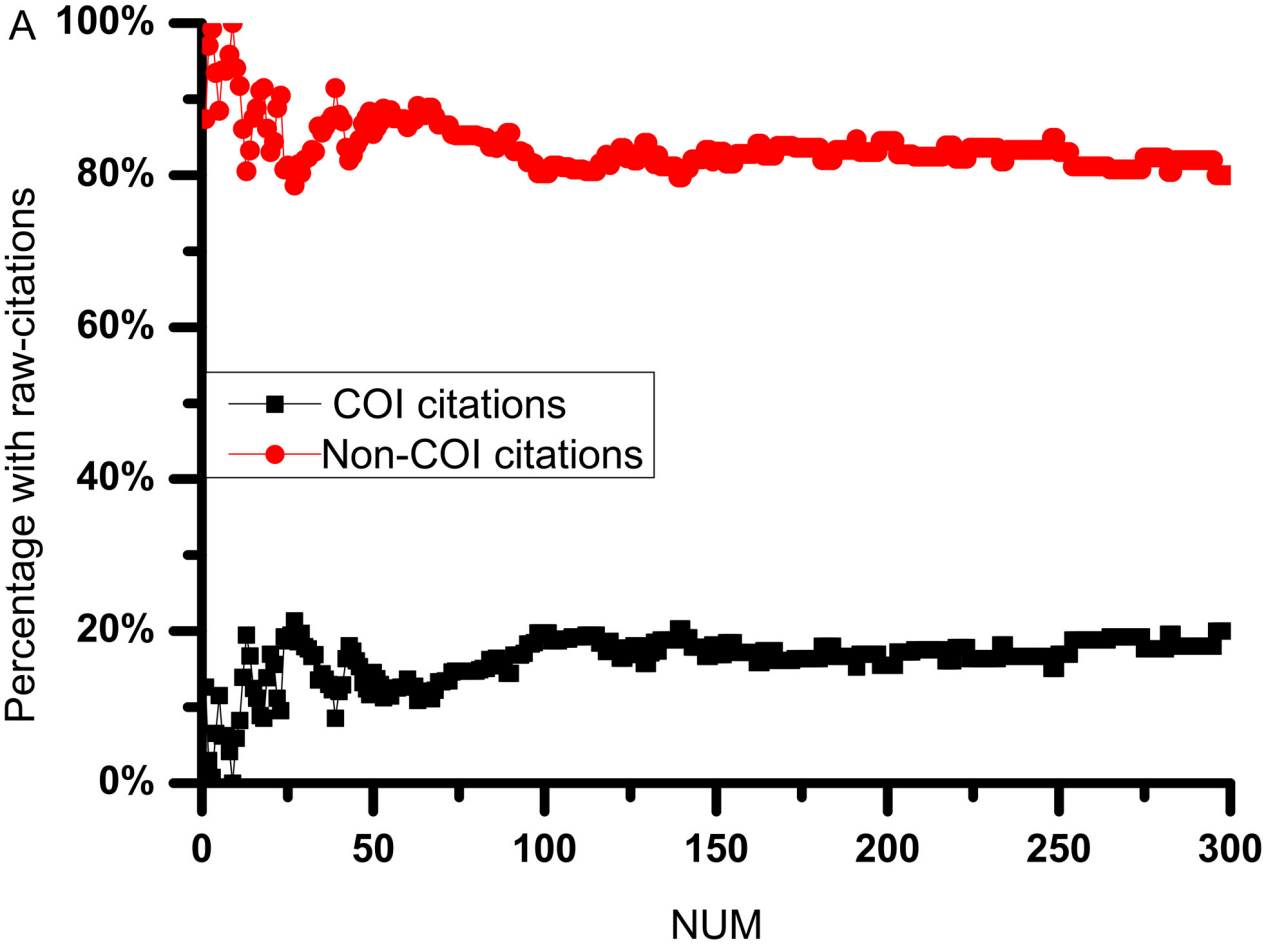
Illustrating the comparison of raw citations, Non-COI citations and COI citations. A: The x-axis indicates journal papers inverse-sorted by citations, and the y-axis indicates the ratios of COI and Non-COI citations over total citations. B: the same experiment with journals exhibiting duplicated patterns of COI citations and Non-COI citations patterns removed.

The comparisons between raw citations and citations without COI (i.e. Non-COI citations) are shown in Figs 4 and 5. The differences between Non-COI citations and raw citations reflect the COI relationship in the citation network. Fig 4 illustrates the citation pattern of the top 10 most cited publications in the dataset. We observe that the number of citations of the top paper is 609, however, among them 237 citations are contributed by their co-authors. We also observed that out of the top 10 cited publications, 4 of them has over 30% self-citations (i.e. cited by co-authors) among their citations. Only one article has not been cited by their co-authors.

Fig 5A compares COI and Non-COI citations patterns for papers with different citation counts, with the x-axis indicates journal papers sorted by descending order of citation counts. We observe that the ratio of COI citations varies significantly for the top 40 journals. Then, we observe a trend of small increases in COI citations ratios when the citation count decreased (i.e. when the inverse-sorted journal number increases.) This observation suggests that for papers with low citation counts, they tend to have a higher number of citations from co-authors or from the same affiliation. The growing COI citations plot suggests that low-cited paper mostly attract COI citations in general. To further elaborate the growth of COI citations, Fig 5B excludes duplicated entries of papers with the same overall and COI citations (i.e. each point represents one or more papers). It was observed for low-cited papers, COI citations can grow beyond Non-COI citations, which demonstrates that some low-cited papers do utilise self-citation or affiliation-citations to as a strategy to boost initial citation counts.

From our observation, we found papers with similar citation counts shares similar anomalous citation patterns. In other words, when the differences of citation count sit between a certain range, the practice of anomalous citations is independent of the manuscript quality. Regarding one specific publication, inappropriate citation activities would gradually become more severe as time goes on. Therefore, removing these aberrant citations are crucial to assess the scientific impact fairly, and our work can be viewed as a exploration by weakening the COI relationship. Furthermore, we compare the performance of COIRank and CAJTRank in terms of Recommendation Intensity (RI) [26]. The essential differences between COIRank and CAJTRank algorithm are the definitions of different citing strength. In CAJTRank algorithm, the citing strengths of all the articles are static, i.e. set to 1. In comparison, in our proposed approach, the citing strength is distinguished by COI relationship, and as time goes on, the citing strength dynamically varies between 0 and 1. In formula (5), the constant *ρ* is set to 0.01 experimentally. To evaluate the performance of different algorithms, we assume that *R* is the list of top *K* returned articles of a ranking approach, *L*_1_ is the list of ground truth, and Non-COI citations are adopted as the ground truth in our method. For one manuscript *P*_*i*_ in *R* with the ranked order *RO*, the RI of *P*_*i*_ at *K* can be defined as:

$$\begin{array}{c} RI\left(P_{i}\right)@K=\left\{\begin{array}{cc} 1+(K-RO)/K & P_{i}\in L_{1}\\ 0 & P_{i}\notin L_{1} \end{array}\right. \end{array}$$ (11)

The above formula denotes that if the manuscript *P*_*i*_ of the top-*K* ground truth list is ranked higher, RI of the manuscript *P*_*i*_ is higher. We can draw the RI of the list *R* at *K* according to the RI of each manuscript. The RI of the list *R* at *K* can be formulized as follows:

$$\begin{array}{c} RI\left(R\right)@K=\sum_{P_{i}\in R}RI\left(P_{i}\right)@K \end{array}$$ (12)

**Fig 6:**
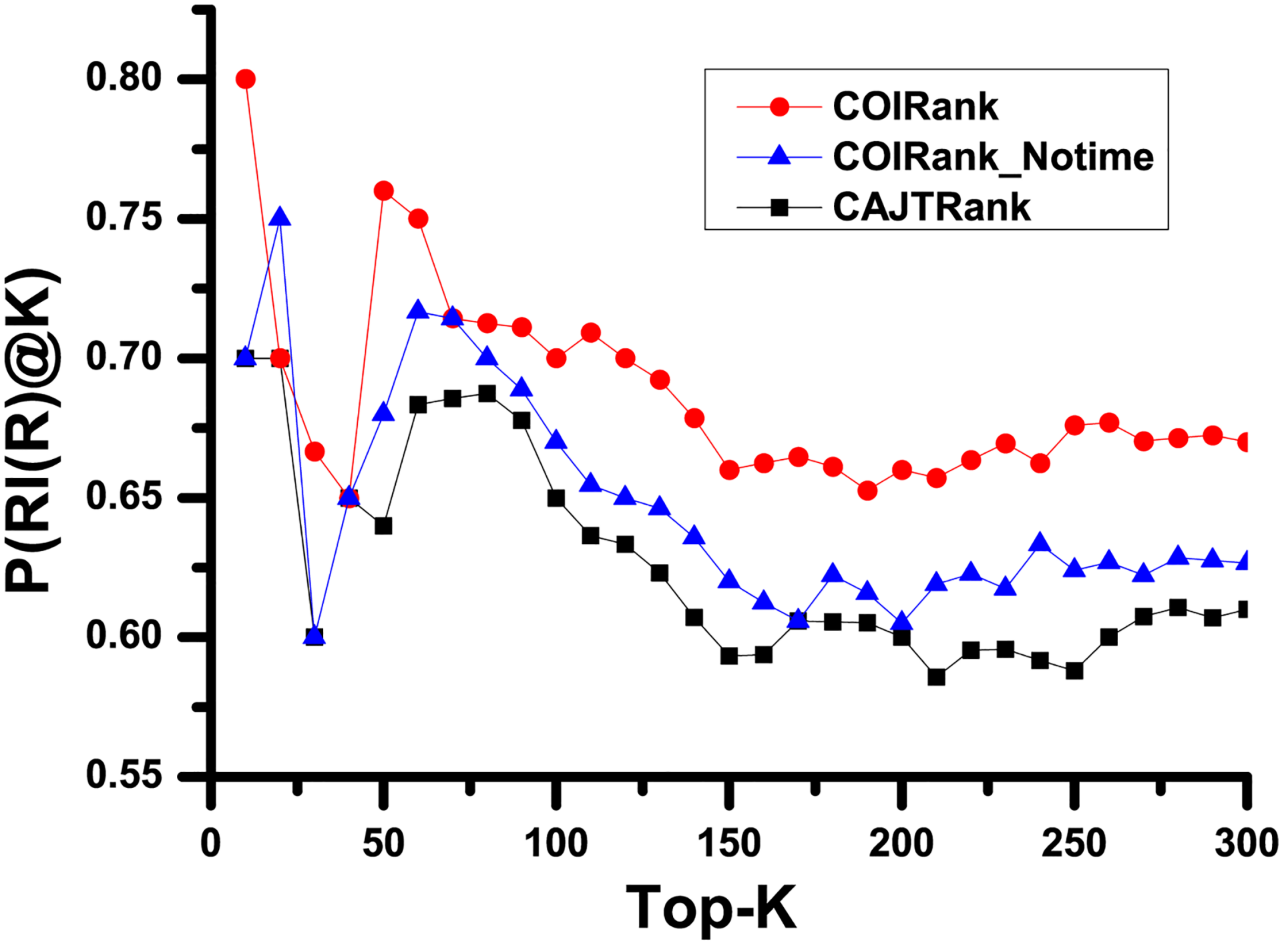
Illustration of the probability of Recommendation Intensity based on different algorithm. The comparison of top-*K* rank results is conducted among time-weighted COIRank algorithm, notime-weighted COIRank algorithm and CAJTRank algorithm. Top-*K* represents *K* papers in the top. *P*(*RI*(*R*)@*K*) is the ratio between *RI*(*R*)@*K* and *K*, namely the precision rate of returned papers.

COIRank and CAJTRank algorithm are tested respectively in the citation network constructed from all the articles in PRC (a subset of APS) journals from 1970 to 2013. Fig 6 depicts the accuracy rate of RI performance of different algorithms. In the overwhelming majority of cases, *P*(*RI*(*R*)@*K*) values of COIRank and COIRank_Notime algorithm are higher than one of CAJTRank algorithm. It’s important to note that when *T*^*Current*^ − *T*^*Cite*^ is used to compute the citing strength $W_{m,n}^{P-Cite}$, the algorithm is called time-weighted algorithm, otherwise, called notime-weighted algorithm. At the same time, time-weighted COIRank algorithm can obtain higher precision than notime-weighted COIRank algorithm except *K* is equal to 20, 40, and 70. The above mentioned results confirm that COIRank outperforms CAJTRank in terms of RI. The main reason behind is shown as follows: On the one hand, we can see that the COIRank algorithm can benefit from weighted PageRank and HITS algorithms. On the other hand, capturing the dynamic evolutionary nature of citation network is useful for rank calculation. Comparing the results of time-weighted method with the corresponding notime-weighted method, it proves that time-weighted method can give a further improvement to the result. The source data of Figs 3–6 is in S1 File.

## Discussion

We proposed the COIRank algorithm to quantify the scientific impact by reproducing the accumulated COI relationship in scientific community. It is known that the core part of citation-based rank metric is PageRank algorithm, which stresses the importance of the citing articles and distributes a high score to the article cited by important articles. In other words, the scores of cited manuscripts enhance with the increasing of citing manuscripts correspondingly. Due to the fact that the citations may be manipulated deliberately, some researchers inflate their research achievement by means of gaining citations from co-authors, friends, colleagues and so on. The prevail of anomalous citations hampers the impartial evaluation of scientific researches, and renders the result that citation-based rank metric does not possess the ability to assess the scientific achievement impartially. The reason is that the PageRank lacks the ability to discriminate mendacious citations. Thus, it is necessary to confront the technical difficulty caused by anomalous citations, i.e. the existence of the COI relationship in the citation network. However, COI and suspected COI should not be ignored for a fair evaluation, and our evaluation method addresses this issue and resolves the limitation of the traditional citation metrics. The presented scheme not only discover the anomalous citations, but also assign a low citing weight to weaken the citation relationship. In addition, COIRank focuses on improving PageRank through setting a weight for PageRank algorithm, and promotes the performance in identifying influential articles.

Since the most outstanding problem is how to define and calculate the citing strength, in order to detect the most determinant factors, we have systemically examined various aspects of citation relationship in our experiment data. Firstly, the previous collaboration relationship between the citing authors and the cited authors has been investigated. Then, the contributed citations by the same affiliations and the co-authors are also surveyed. We find that the COI relationship is crucial to deal with above mentioned problem. Without loss of generality, we consider each pair authors’ COI relationship between the citing article and the cited article. If each pair authors have not collaborated with each other before, and they belong to the same affiliation, we believe they have suspected COI relationship, which is one of our significant contributions. Based on the above considerations, times of collaboration, time span of collaboration, times of citing and time span of citing are composed in our scheme to decide the citing strength. In terms of citing strength, a basic idea is that the COI relationship between the citing publication and the cited publication is more serious, thus the citing strength should be set to a lower value. In particular, such processing constrains the negative effects by anomalous citations, and guarantees that the scores of articles are updated in each PageRank iteration process impartially.

We implement a multivariate linear regression to estimate the parameters of the COIRank, COIRank_Notime, and CAJTRank algorithms. Measuring the impact of a scholarly manuscript relies on the scores of its PageRank, authors, journal, and references. We found that *δ* obtained a very small value, approximately equal to 0.01, indicating that the scores of references are not very important compared with other factors. However, *α*, *β* and *δ* parameters constrained each others and played crucial roles in terms of RI. Through observing the results of returned RI by using these parameters, we estimated *α* = 0.15 ± 0.05 and *β* = 0.30 ± 0.10, indicating a significant relative RI increase. The optimal parameters are used for all the aforementioned methods.

In fact, the COIRank approach aims to measure the impact of individual publication, while unbiased appraisal to publications are the foundation for other scientific entities, ranging from authors to journals, teams, affiliations and even entire countries. In this competitively academic environment, promotion of individuals, funding, survival of teams and affiliations depend on the scientific impact of their publications fundamentally. Our proposed method is promising and can be considered complementary to the existing citation metrics, such as IF, H-index, and g-index.

Since COI relationship contains many factors, the objective of our future research is to evaluate the impact of publication more accurately. For example, we plan to mine more COI relationships, such as the relationship between teachers and students, friend relationship, community relationship and so on. We believe that the strength of citation relationship should be recommended to a small value between teachers and students or between friends. The strength of the citation relationship between each two authors is stronger in different communities than that in the same community. The explorations of the impact of scholarly article of different affiliations and countries based on COI relationship are also part of our future work.

## Supporting Information

S1 File The source data of Figs 3–6. (XLSX)
